# Impact of egg incubation on hemocyte recruitment and susceptibility of *Galleria mellonella* larvae to pathogens

**DOI:** 10.3389/fmicb.2025.1611104

**Published:** 2025-06-03

**Authors:** Paulo Henrique Fonseca Carmo, Patrícia Michelle Nagai de Lima, Fabiana Alves Silva, Jaqueline Lemes Ribeiro, Juliana Campos Junqueira, Maíra Terra Garcia

**Affiliations:** ^1^Departamento de Biociências e Diagnóstico Bucal, Instituto de Ciência e Tecnologia, Universidade Estadual Paulista (UNESP), São José dos Campos, Brazil; ^2^Department of Genetics, Microbiology and Immunology, Institute of Biosciences, Universidade Estadual Paulista (UNESP), Botucatu, Brazil; ^3^Universidade Mogi das Cruzes, Mogi das Cruzes, Brazil

**Keywords:** *Galleria mellonella*, colony cultivation, incubation, breeding, egg activation

## Abstract

**Introduction:**

*Galleria mellonella* is widely used as an *in vivo* model in microbiological and toxicological studies due to its reproducibility, low cost, and ease of handling. However, maintaining homogeneous laboratory colonies presents challenges. This study evaluated whether different incubation methods influence the larval immune response against *Staphylococcus aureus, Candida albicans*, and *Escherichia coli*.

**Methods:**

Four experimental groups were established: (1) Traditional group (TG), eggs stored at 27°C; (2) Immediate group (IG), eggs stored at 16°C for 120 days before incubation at 27°C; (3) Gradual group (GG), temperature gradually reduced from 27°C to 16°C over 98 days, then increased back; and (4) Frozen group (FG), eggs stored at freezing temperatures.

**Results and discussion:**

FG eggs were non-viable, and IG and GG showed delayed development. IG larvae were more susceptible to infections, while GG larvae resembled TG. Thus, GG can serve as an effective alternative rearing method.

## Graphical abstract

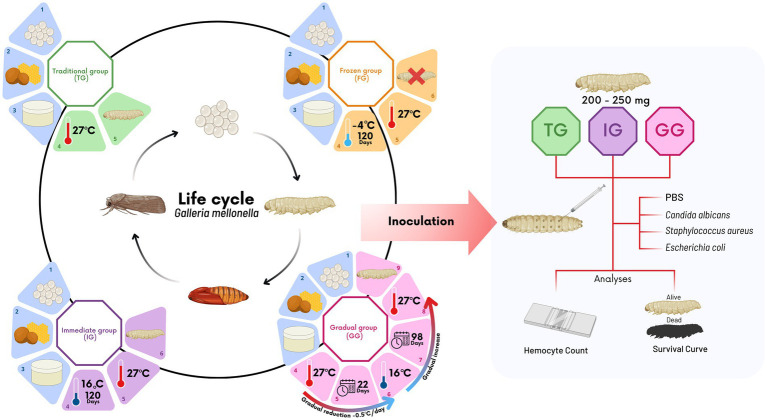

## 1 Introduction

Understanding human infections and host-pathogen interactions relies heavily on research using animal models. Each year, millions of vertebrates are used in scientific experiments, with mice and rats being the most used species (Mukherjee et al., 2022). This practice has come under increasing scrutiny, leading to stricter ethical regulations and longer approval processes. The high costs and logistical complexities associated with vertebrate models have prompted researchers to explore alternative models, such as insects. Among these, *Galleria mellonella*, commonly known as the greater wax moth, has become an increasingly valuable alternative *in vivo* model in microbiological and toxicological research (Kling, 2020; Gallorini et al., 2024) due to its susceptibility to a wide range of human pathogens, including bacteria (e.g., *Staphylococcus aureus*) (Lyu et al., 2017; Zhao et al., 2019; Guevara et al., 2022; Ten et al., 2023; Gallorini et al., 2024; Yu et al., 2024) and fungi (e.g., *Candida albicans*) (Mylonakis et al., 2005; Eisenman et al., 2014; Benaducci et al., 2016; Rossoni et al., 2017; Jin et al., 2020; Meccatti et al., 2022; Jothi and Gowrishankar, 2024).

This invertebrate offers significant advantages over vertebrate models, including reduced costs, easier handling compared to other insects commonly used as alternative *in vivo* models (e.g., *Drosophila melanogaster*), and good adaptability to human physiological temperature (37°C) and laboratory conditions, which influence microbial virulence factors. Additionally, *G. mellonella* is suitable for large-scale studies and does not require ethical approval (Mukherjee et al., 2011; Kling, 2020; Ten et al., 2023; Gallorini et al., 2024). The immune system of *G. mellonella* shares significant similarities with the mammalian innate immune system, which enhances its reliability as an *in vivo* model in microbiological research (Ten et al., 2023; Gallorini et al., 2024). This similarity includes mechanisms such as phagocytosis by hemocytes and the production of antimicrobial peptides (Tsai et al., 2016; Gallorini et al., 2024). Further, the production of cytokine-like molecules (e.g., interleukins, interferon and Tumor necrosis factor) by *G. mellonella* increased after stimulation by fungal infection, suggesting their capacity of modulating their immune responses (Wrońska et al., 2024). These features indicate *G. mellonella’*s potential to elucidate mammalian immune responses to infections, making it a valuable *in vivo* model for studying microbial pathogenesis, host-pathogen interactions, and the efficacy and toxicity of antimicrobial treatments (Mukherjee et al., 2011; Champion et al., 2016; Rossoni et al., 2017; Andrea et al., 2019; Bugyna et al., 2023; Ten et al., 2023).

Despite these advantages, obtaining and maintaining *G. mellonella* can pose significant challenges. The limited commercial availability of *G. mellonella* with a specific genotype, raised under standardized conditions, restricts accessibility for many laboratories. Typically, larvae are sourced from independent breeders, resulting in an uncontrolled environment that leads to inconsistencies in genotypes, breeding conditions and maintenance (Mylonakis, 2008; de Jong et al., 2022). These variations can influence the quality, health and their susceptibility to infections, negatively affecting the consistency and reproducibility of experimental results (Tsai et al., 2016). A solution that different laboratories adopt to address these challenges is the in-lab rearing of *G. mellonella*.

Although the rearing of *G. mellonella* in the laboratory is considered simpler than the breeding and maintenance of vertebrates and even other invertebrates, it still poses significant challenges and requires substantial dedication from the team involved (Giammarino et al., 2024). Keeping these larvae in optimal conditions demands continuous and meticulous work, including thorough cleaning of the containers where the larvae are kept, as any lapse in this process can compromise the colony’s health and render it unusable for experiments. Additionally, monitoring food, humidity, and temperature is crucial to ensure the proper development of the larvae, as even slight variations can negatively impact their life cycle (Pereira et al., 2020).

Due to the cyclical nature of *G. mellonella* rearing and the difficulty of obtaining eggs regularly, laboratories that choose to maintain these larvae often need to sustain them continuously, even when the larvae are not being directly used in experiments. However, maintaining uninterrupted rearing can become a significant challenge, due the high consumption of materials, such as substrate and food, along with the time and effort required from the team to ensure optimal hygiene and management conditions. This reality places many laboratories in a difficult position (Jorjão et al., 2018b; de Jong et al., 2022).

Thus, this study evaluated the effects of different egg incubation methods on larval susceptibility to various pathogens (*S. aureus, E. coli*, and *C. albicans*), including the hemocyte response to these infections. By addressing these issues, we sought to facilitate larval rearing in the laboratory and the use of *G. mellonella* in experimental studies, ensuring more consistent results across studies and laboratories.

## 2 Materials and methods

### 2.1 Experimental groups

**Figure 1:**
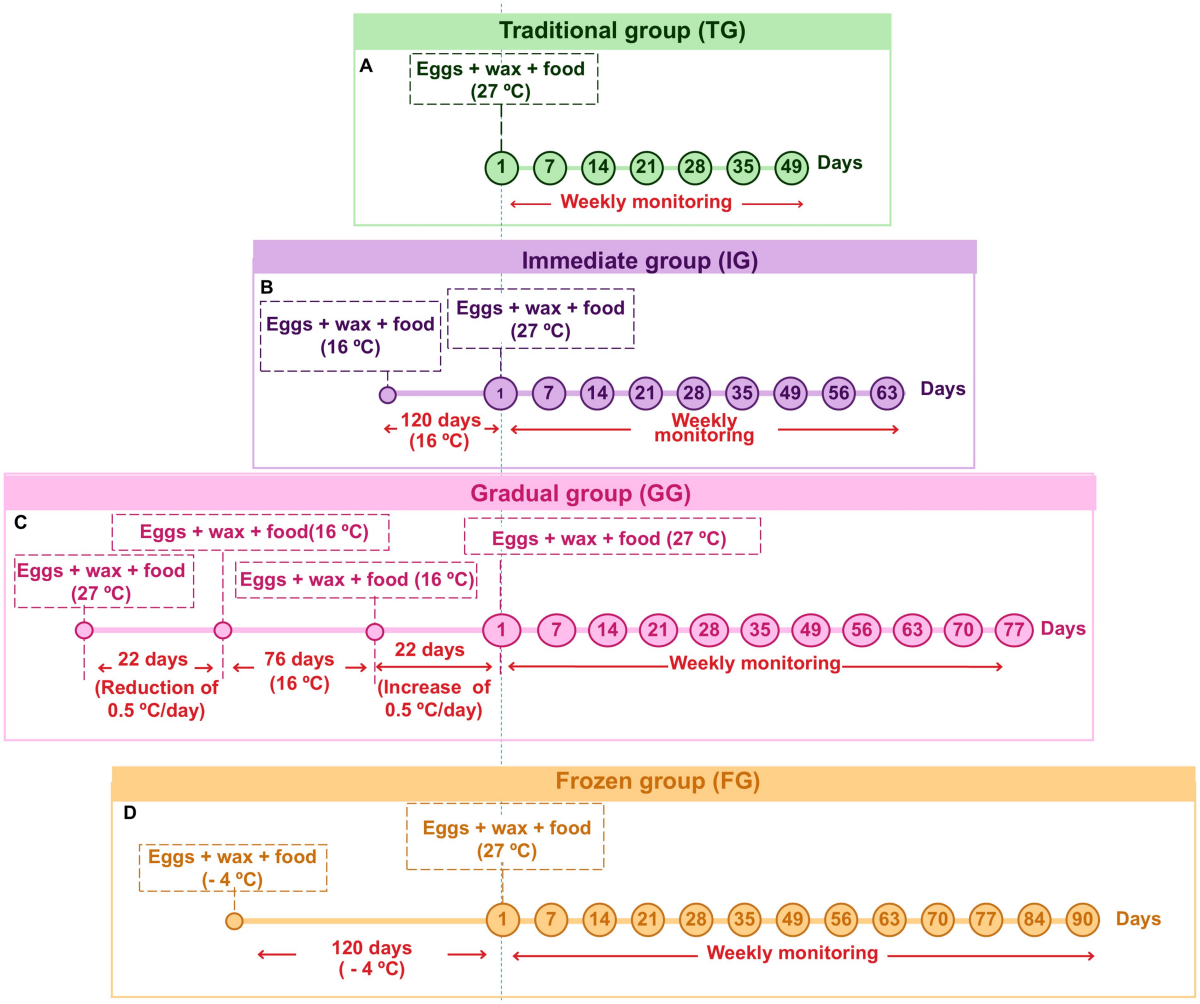
Timeline of experimental parameters and incubation conditions applied to the eggs in the traditional (TG) **(A)**; immediate (IG) **(B)**; gradual (GG) **(C)**; and frozen (FG) **(D)** groups during weekly monitoring.

In this study, four experimental groups were designed to simulate different conditions for maintaining and activating *G. mellonella* eggs: traditional, immediate, gradual, and frozen. A more detailed schematic of these conditions is presented in Figure 1. Eggs collection across the four experimental groups was conducted uniformly. Colored paper containing eggs recently laid by the female moths created in the invertebrate laboratory of the Microbiology and Immunology laboratory of ICT/Unesp., using the conventional protocol (Jorjão et al., 2018b) was collected and divided into four equal parts, with each part assigned to one of the experimental groups. Since the quantity and quality of food significantly affect larval weight, fecundity, and overall growth rates (Champion et al., 2018; Firacative et al., 2020; Hickin et al., 2021), all groups were fed with the same protein-rich diet (Jorjão et al., 2018b). Furthermore, the relative humidity and photoperiod conditions were maintained consistently across all experimental groups (Jorjão et al., 2018b). However, different methodologies for the incubation and conditioning of the eggs were employed for each experimental group.

In addition, images of each experimental group were taken every 7 days using a Zeiss Stemi 2000-C Stereo Microscope. Images of intact, hatched, or damaged eggs were captured at 16x magnification, while images of larvae and pupae were captured at 6.5x.

#### 2.1.1 Traditional group (TG)

In TG, the conventional egg conditioning protocol was followed (Jorjão et al., 2018b), which has been used by our laboratory for over 10 years. After collecting the eggs, they were placed in a matte white plastic container along with the wax and feed. The container was then incubated at 27°C and monitored weekly until the eggs hatched.

#### 2.1.2 Immediate group (IG)

In this group, the temperature was abruptly changed. After collecting the eggs, they were placed, along with the wax and feed, in a matte white plastic container. The container was then incubated at 16°C and maintained at this temperature for 120 days and monitored weekly. Following this period, the eggs were incubated at 27°C and monitored weekly to check for larval hatching. This group represents the urgent need to interrupt the rearing process by storing the eggs at low temperatures for extended periods.

#### 2.1.3 Gradual group (GG)

In the GG, the temperature was initially decreased and then gradually increased. After the eggs were collected, they were placed in a matte white plastic container along with wax and feed. The container was initially incubated at 27°C, with a gradual reduction of 0.5°C per day (for easier adaptation to temperature changes) until it reached 16°C over 22 days. The eggs were then maintained at 16°C for an additional 76 days, totaling 98 days of incubation, with weekly monitoring. After the incubation period, the temperature was gradually increased by 0.5°C daily up to 27°C, totaling another 22 days, resulting in a final cycle of 120 days, with daily monitoring for signs of hatching. Once the temperature stabilized at 27°C, weekly monitoring was continued to observe larval hatching.

This group represents a planned strategy involving the gradual reduction of temperature, followed by long-term storage at lower temperatures. The culture is then reactivated by gradually increasing the temperature to ideal experimental conditions.

#### 2.1.4 Frozen group (FG)

In the FG, the eggs were initially frozen and then reactivated. For this, after collection, the eggs were placed in a matte white plastic container along with the wax and feed. The container was then stored in a freezer at −4°C for 120 days. Following this period, food was provided for the eggs, and the temperature was raised to 27°C. The eggs were monitored weekly for 3 months to check for larval hatching. This group was created to simulate an attempt to abruptly stop the culture by freezing the eggs. The culture was then reactivated by rapidly raising the temperature to the ideal experimental conditions.

### 2.2 Evaluation of the effects of different egg incubation methods on the response of *G. mellonella* to infections by *Candida albicans*, *Escherichia coli* and *Staphylococcus aureus*

After the eggs from the different experimental groups hatched, *G. mellonella* larvae were conventionally fed with feed and wax, that is, three times a week the larvae were cleaned, and the food was replaced with fresh food. Then, 15 larvae (per group) weighing between 200 and 250 mg were selected to evaluate the impacts of egg storage conditions on hemocyte recruitment and the susceptibility of larvae to experimental infection. Larvae from TG, IG and GG were divided into 5 groups: (i) without intervention, (ii) inoculation with PBS, (iii) inoculation with *Candida albicans*, (iv) inoculation with *Escherichia coli*, and (v) inoculation with *Staphylococcus aureus*.

#### 2.2.1 Microorganism cultivation

We used reference strains of *Candida albicans* ATCC 18804, *Escherichia coli* ATCC 25922, and *Staphylococcus aureus* ATCC 6538. *Candida albicans* was cultivated on Sabouraud dextrose agar (SDA; Kasvi, Pinhais, Brazil), while *E. coli* and *S. aureus* were cultivated on Mueller–Hinton (MH; Himedia, Mumbai, India) agar and Brain Heart Infusion (BHI) agar (Kasvi), respectively. All cultures were incubated for 48 h at 37°C.

#### 2.2.2 Survival curves of *G. mellonella*

The susceptibility of *Galleria mellonella* larvae to microbial infection was evaluated using survival curve assays. Isolated colonies of *C. albicans*, *E coli*, and *S. aureus*, previously grown for 48 h at 37°C on SDA, MH and BHI agar, respectively, were transferred to BHI broth (Kasvi) for *S. aureus* and *E. coli* strains, and to Yeast Peptone Dextrose (YPD; Difco, Detroit, MI, USA) broth for *C. albicans*, followed by incubation at 37°C for 24 h. After this period, microbial suspensions were prepared in PBS and all microorganisms were standardized to 10^8^ cells/mL. Then, the larvae were inoculated with 10 μL of PBS (control group), *C. albicans*, *E. coli* or *S. aureus*, according to each experimental group, by injection into the last right proleg using a 10 μL 26G Hamilton syringe (Hamilton Company, Reno, Nevada, USA). A control group of non-infected (NI) larvae was included. Each experimental group consisted of 15 randomly selected larvae. Survival rates were monitored every 24 h for over a period of 7 days. The larvae were considered dead when no movement was observed after gentle prodding (Ribeiro et al., 2017).

#### 2.2.3 Hemocyte recruitment

For hemocyte recruitment, the hemolymph was collected 6 h after inoculation. The larvae were immobilized in an ice-cold Petri dish for 30 min and a small incision was made in the ventral side in a cephalocaudal direction using a scalpel. Subsequently, the hemolymph was removed, transferred to ice-cold microtubes containing isotonic phosphate solution [IPS; 2% sodium chloride, 100 mM glucose, 30 mM sodium citrate, 10 mM ethylenediaminetetraacetic acid salt dihydrate (EDTA), 26 mM citric acid monohydrate] medium in a ratio of 1:99 (hemolymph:IPS), and then 10 μL were placed in the hemocytometer for hemocyte quantification (Figueiredo-Godoi et al., 2019). At least four fields were analysed for each larval hemolymph sample.

### 2.3 Statistical analysis

The hemocyte recruitment was statistically analyzed using one-way analysis of variance (ANOVA), followed by Tukey’s test. Statistical analysis of *G. mellonella* survival curve was performed using the log-rank test (Mantel-Cox). All analysis were performed using GraphPad Prism 6 Program (GraphPad Software, Inc., La Jolla, CA, USA). Values of *p* < 0.05 were considered statistically significant.

## 3 Results

### 3.1 Effects of the egg conditioning method on TG larvae

**Figure 2:**
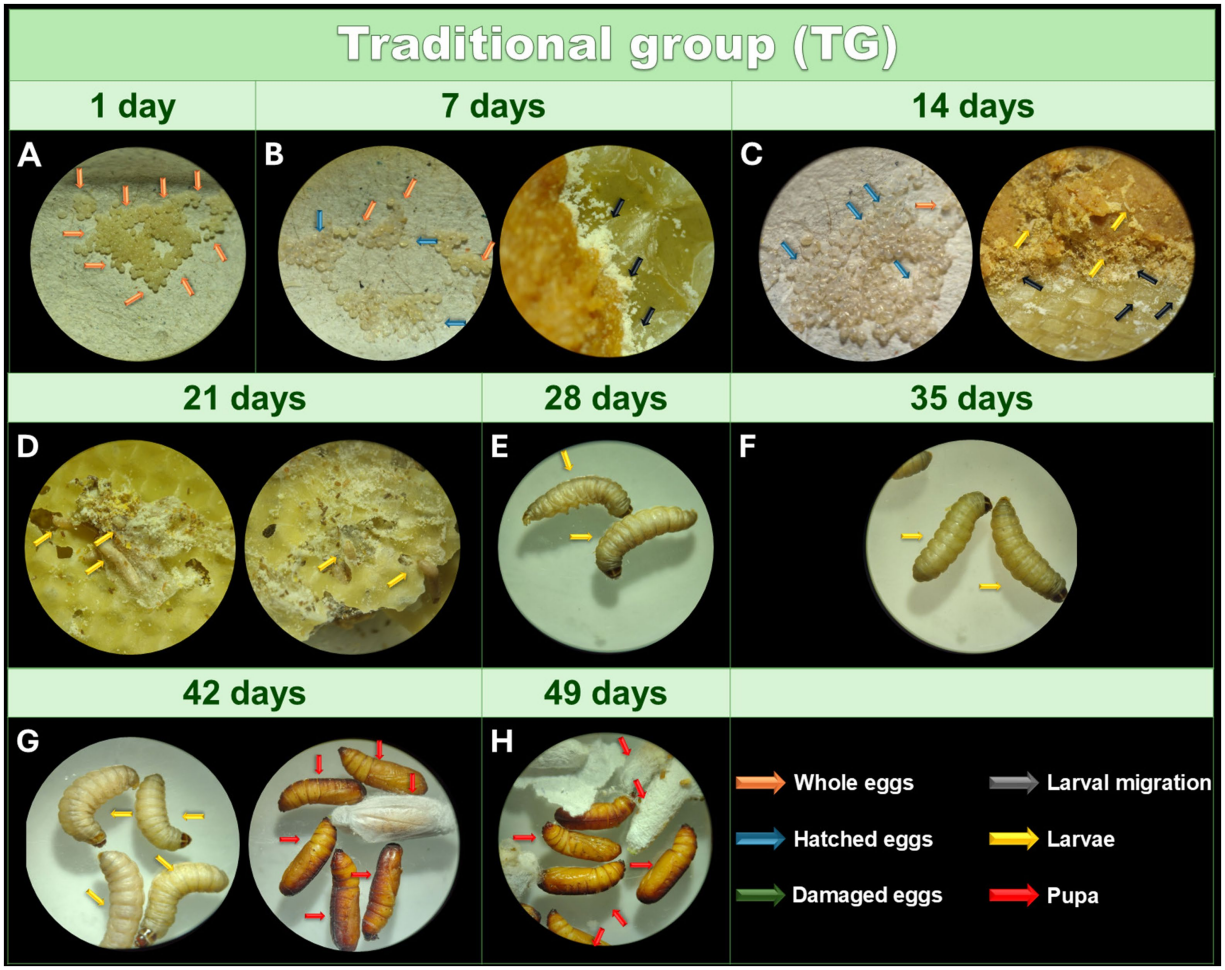
Monitoring of the development of *Galleria mellonella* eggs from the TG. Monitoring of the development of *G. mellonella* eggs from the traditional group (TG) over 49 days. Image (at magnification of 16x and 6.5x) sequence shows the developmental stages of *G. mellonella*, from eggs to pupae formation. The TG was monitored at 1 **(A)**, 7 **(B)**, 14 **(C)**, 21 **(D)**, 28 **(E)**, 35 **(F)**, 42 **(G)**, and 49 **(H)** days.The recorded stages include eggs, larvae and pupae. The evolution of morphological characteristics at each stage are indicated with arrows of different colors. Orange: intact eggs; blue: hatched eggs; green: damaged eggs; black: larval migration; yellow: adult larvae; and red: pupa.

In Figure 2A it is possible to observe the eggs recently collected from the TG. After the incubation period, eggs hatched and transferred to wax with food 7 days after collection. However, analysis by optical microscopy did not reveal larvae (Figure 2B). After 14 days, most of the eggs hatched and larvae were observed moving through the feed and wax under a microscope (Figure 2C). However, during this period, handling the contents is still not recommended to avoid damaging the newly hatched larvae. After 21 days, the larvae were visible to the naked eye (Figure 2D). At this stage, the containers where the larvae are kept must be cleaned to prevent dirt and moisture from accumulating. The larvae reached the penultimate larval instar after 28 days (Figure 2E). The last larval instar and weight required for experimental use (200–250 mg) were achieved between 35 and 42 days (Figure 2F). The first pupae emerged after 42 days (Figure 2G), and all larvae had transformed into pupae after 49 days (Figure 2H). During the analysis period, 95% of the eggs hatched and the larvae were intact, active, with excellent cocoon formation, and without dark pigments or striking lines.

### 3.2 Effects of the egg conditioning method on IG larvae

**Figure 3:**
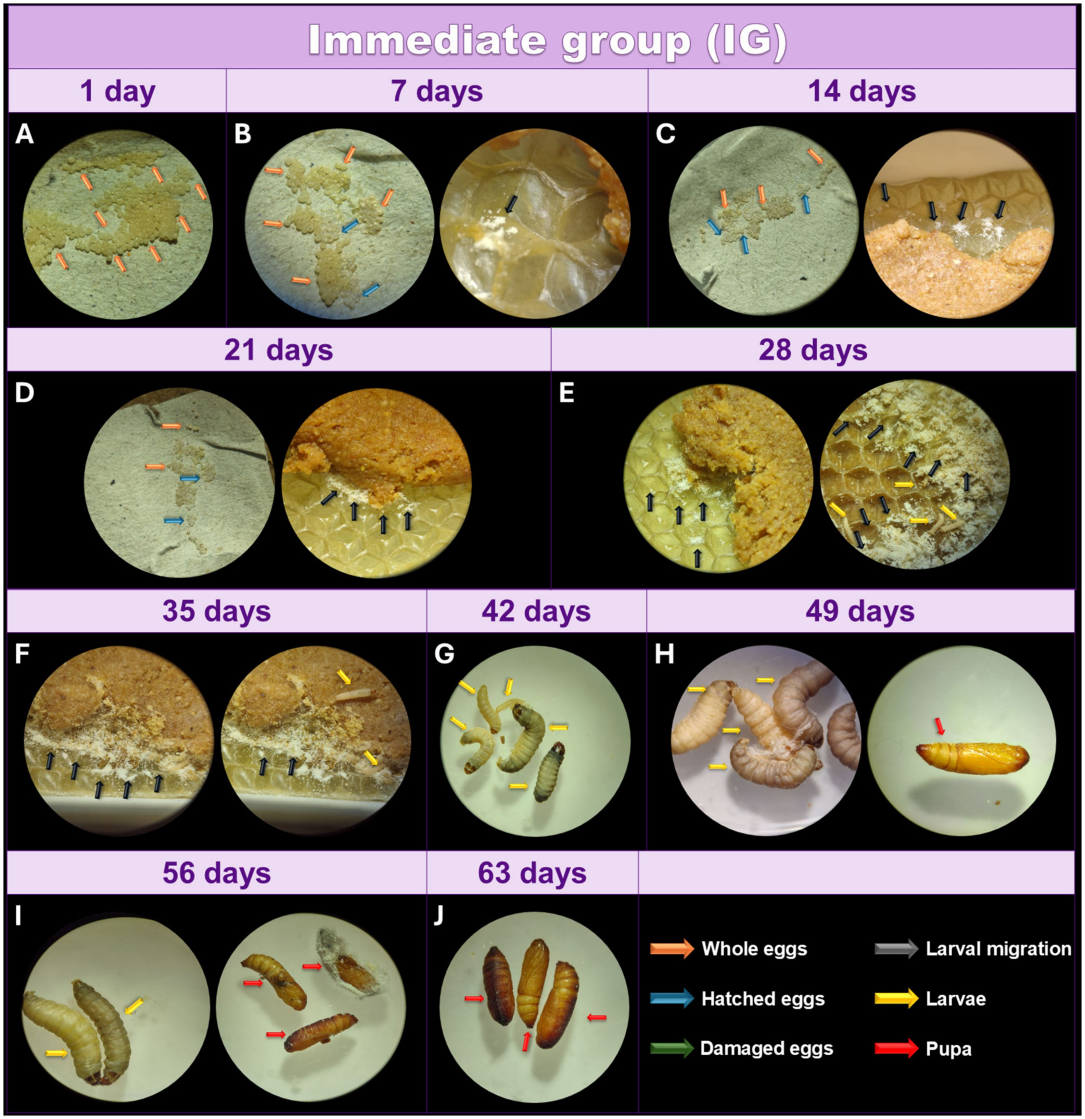
Monitoring of the development of *Galleria mellonella* eggs from the IG. Monitoring of the development of *G. mellonella* eggs from the immediate group (IG) over the days. Image (at magnification of 16x and 6.5x) sequence shows the developmental stages of *G. mellonella*, from eggs to pupae formation. The IG was monitored at 1 **(A)**, 7 **(B)**, 14 **(C)**, 21 **(D)**, 28 **(E)**, 35 **(F)**, 42 **(G)**, 49 **(H)**, 56 **(I)**, and 63 **(J)** days. The recorded stages include eggs, larvae and pupae. The evolution of morphological characteristics at each stage are indicated with arrows of different colors. Orange: intact eggs; blue: hatched eggs; green: damaged eggs; black: larval migration; yellow: adult larvae; and red: pupa.

After collecting (Figure 3A), the eggs were incubated at 27°C, and the day count began immediately. During the first 7 days, 95% of the eggs remained intact, while a small percentage hatched, with the larvae transferring between the feed and the wax (Figure 3B). At this stage, the larvae clustered together and looked like a yellowish-white powder. This powdery appearance signals the migration of first-instar larvae toward the food source. However, they remain undetectable even under optical microscopy, as they are embedded within the wax and feed. However, these larvae were not yet visible under the optical microscope at the magnification used (16X). After 14 days, approximately 50% of the eggs remained intact, and the hatched larvae showed slow growth, still represented by something similar to a yellowish-white powder under optical microscopy (Figure 3C).

After 21 days, 80% of the eggs had hatched (Figure 3D), while the remaining 20% did not hatch by the 28-day mark, suggesting a lack of viability (Figure 3E). At this stage, some larvae were observed under the microscope, while most still appeared as a yellowish-white powder, indicating reduced metabolism compared to the TG. While some larvae displayed greater growth at 35 days, others remained very small (Figure 3F). After 42 days, some adult larvae were visible, while others remained at an early larval stage (Figure 3G). Although all larvae were active and intact, some displayed different coloring with well-defined lines, suggesting compromised health. The first pupae appeared after 49 days (Figure 3H) however, at 56 days there were still larvae and pupae (Figure 3I), and complete pupation was observed after 63 days (Figure 3J).

### 3.3 Effects of the egg conditioning method on GG larvae

**Figure 4:**
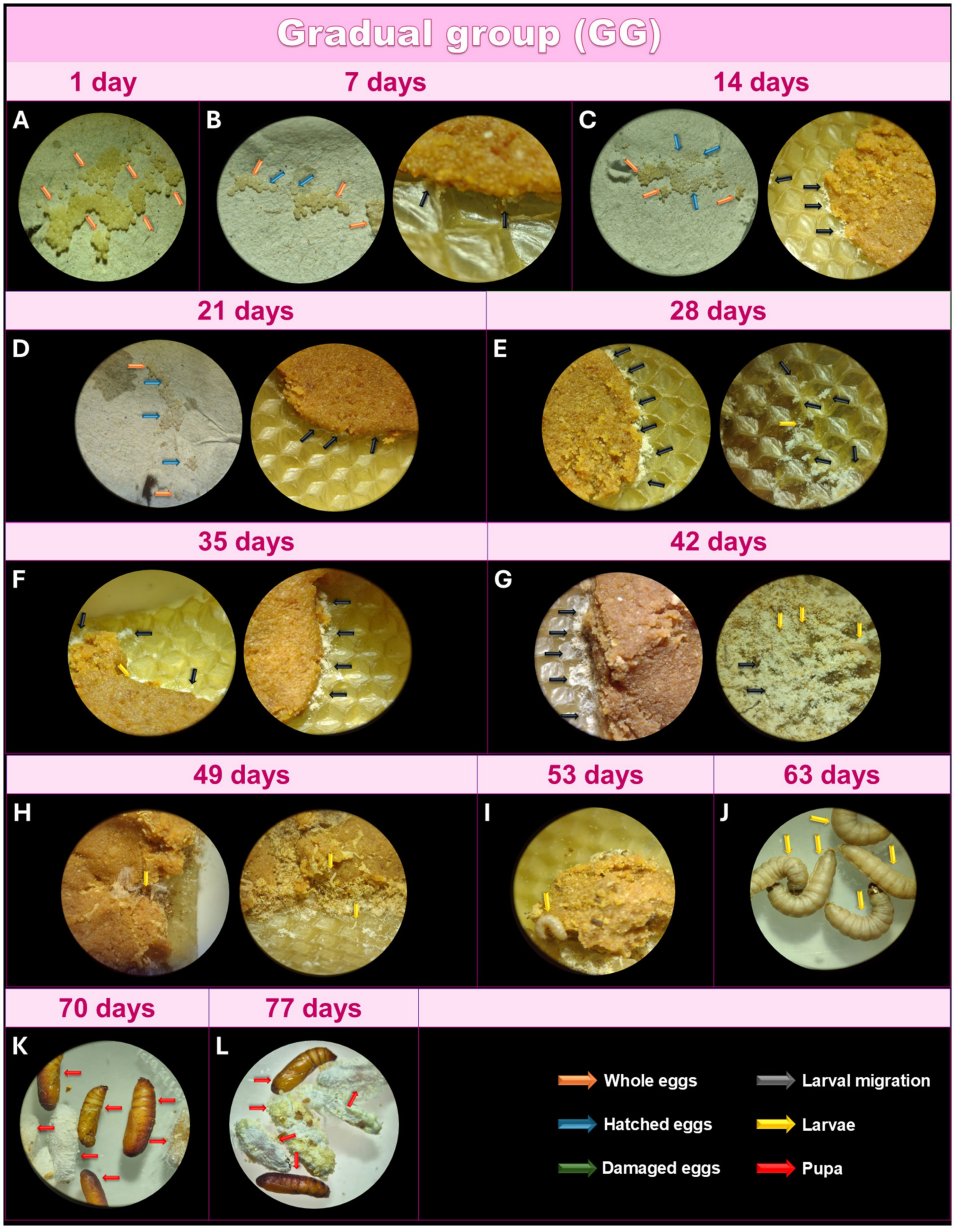
Monitoring of the development of *Galleria mellonella* eggs from the GG. Monitoring of the development of *G. mellonella* eggs from the gradual group (GG) over the days. Image (at magnification of 16x and 6.5x) sequence shows the developmental stages of *G. mellonella*, from eggs to pupae formation. The GG was monitored at 1 **(A)**, 7 **(B)**, 14 **(C)**, 21 **(D)**, 28 **(E)**, 35 **(F)**, 42 **(G)**, 49 **(H)**, 56 **(I)**, 63 **(J)**, 70 **(K)** and 77 **(L)** days. The evolution of morphological characteristics at each stage are indicated with arrows of different colors. Orange: intact eggs; blue: hatched eggs; green: damaged eggs; black: larval migration; yellow: adult larvae; and red: pupa.

After collecting the eggs (Figure 4A), they were incubated, and in this group, the day count began when the eggs were stored at 27°C. During the first 7 days, 97% of the eggs remained intact, while a small percentage hatched, and the larvae moved toward the feed (Figure 4B). At this stage, the larval structure was not yet visible under optical microscopy at 16X magnification; only a material resembling a yellowish-white powder could be observed. After 14 days, 85% of the eggs had not hatched, and the newly hatched larvae displayed slow growth compared to TG (Figure 4C). After 21 days, larvae continued to show slow growth, with approximately 60% of the eggs still unhatched (Figure 4D). After 28 days, 95% of the eggs had hatched, while 5% were still intact, indicating nonviability of those eggs (Figure 4E). At this point, the larval morphology of some was visible under light microscopy, but most remained as a yellowish-white powder, exhibiting remarkably slower growth than TG but similar to IG. A few more developed larvae were observed between 35 and 42 days (Figures 4F,G), though most retained their yellowish-white powdery appearance. After 49 days, the larvae began to grow, reaching a size visible to the naked eye (Figure 4H). Adult larvae were observed after 56 days (Figure 4I), and they reached a size suitable for the experiment after 63 days (Figure 4J). The first pupae were seen after 70 days (Figure 4K), with all larvae transforming into pupae by 77 days (Figure 4L). Like the TG, the larvae in the GG were intact, active, free of dark pigments or lines, and exhibited excellent cocoon formation at the appropriate stages.

### 3.4 Effects of the egg conditioning method on FG

In this group, the eggs were monitored for 90 days but did not hatch, indicating that the conditioning method likely hindered the eggs’ ability to hatch and prevented the subsequent larval growth.

In addition, the effects of egg conditioning in different groups were summarized in the Table 1.

**Table 1:** Effects of egg conditioning methods – traditional group (TG), immediate group (IG), gradual group (GG) and frozen group (FG) – on the percentage (%) of egg, larvae and pupae of *Galleria mellonella* during weekly monitoring.

Group	Stage	Days												
		7	14	21	28	35	42	49	56	63	70	77	84	90
TG	Egg	50%	0%	0%	0%	0%	0%	0%	EC	EC	EC	EC	EC	EC
	Larvae	0% (Not visible under optical microscopy)	100% (Visible under optical microscopy)	100% (Visible to the naked eye)	100% (Visible to the naked eye)	100% (Larvae in the last larval instar)	90% (Larvae in the last larval instar)	0%	EC	EC	EC	EC	EC	EC
	Pupa	0%	0%	0%	0%	0%	10%	100%	EC	EC	EC	EC	EC	EC
IG	Egg	95%	50%	20%	20%	20%	20%	20%	20%	20%	EC	EC	EC	EC
	Larvae	5% (Not visible under optical microscopy)	50% (Not visible under optical microscopy)	80% (Visible under optical microscopy)	80% (Visible under optical microscopy)	80% (Partially visible under optical microscopy and to the naked eye)	80% (Partially visible under optical microscopy and to the naked eye)	70% (Larvae in the last larval instar)	30% (Larvae in the last larval instar)	0%	EC	EC	EC	EC
	Pupa	0%	0%	0%	0%	0%	0%	10%	50%	80%	EC	EC	EC	EC
GG	Egg	97%	85%	60%	5%	5%	5%	5%	5%	5%	5%	5%	EC	EC
	Larvae	3% (Not visible under optical microscopy)	15% (Not visible under optical microscopy)	40% (Not visible under optical microscopy)	95% (Visible under optical microscopy)	95% (Visible under optical microscopy)	95% (Visible under optical microscopy)	95% (Partially visible under optical microscopy and to the naked eye)	95% (Visible to the naked eye)	95% (Larvae in the last larval instar)	85% (Larvae in the last larval instar)	0%	EC	EC
	Pupa	0%	0%	0%	0%	0%	0%	0%	0%	0%	10%	95%	EC	EC
FG	Egg	100%	100%	100%	100%	100%	100%	100%	100%	100%	100%	100%	100%	100%
	Larvae	0%	0%	0%	0%	0%	0%	0%	0%	0%	0%	0%	0%	0%
	Pupa	0%	0%	0%	0%	0%	0%	0%	0%	0%	0%	0%	0%	0%

TG, traditional group; GG, gradual group; IG, immediate group; FG, frozen group; EC, end of cycle.

### 3.5 Effects of egg conditioning method on survival after infection

**Figure 5:**
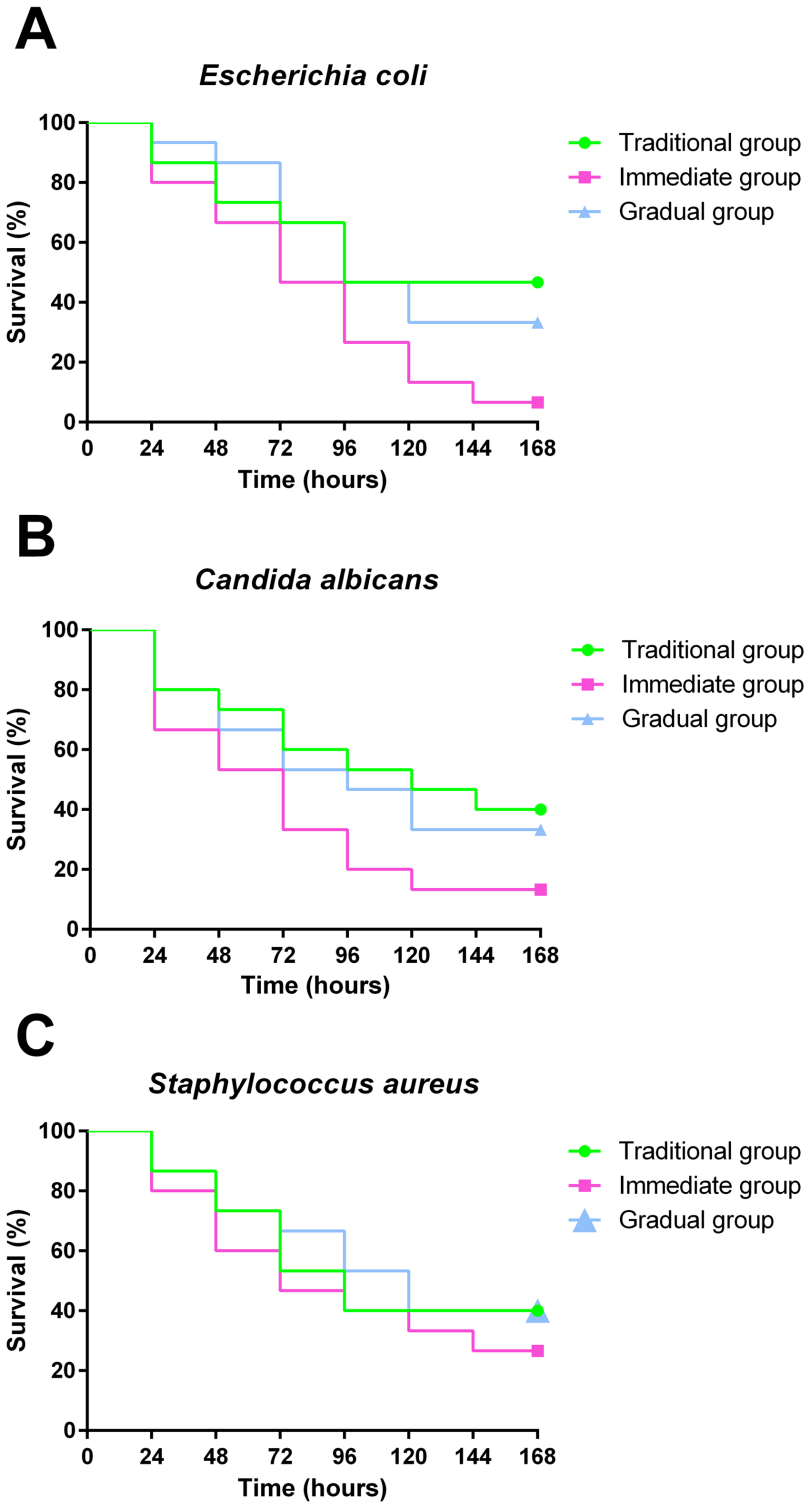
Survival curves after infection. Survival curve of *G. mellonella* from groups under different conditions: Traditional group (TG - green), Immediate group (IG - pink) and Gradual group (GG - blue), following infection with *E. coli* **(A)**, *C. albicans* **(B)** and *S. aureus* **(C)**, for evaluation of the susceptibility of larvae to microbial infection. Statistical analyses were performed using the log-rank test (Mantel-Cox) (*p* < 0.05).

Larvae infected with *E. coli* exhibited survival rates of 46.6, 6.6, and 33% for TG, IG, and GG, respectively (Figure 5A). The survival rates after infection with *C. albicans* were 40% in TG, 13.3% in IG and 33% in GG (Figure 5B). The infection of larvae with *S. aureus* resulted in survival rates of 40, 26 and 40% for TG, IG and GG, respectively (Figure 5C). Non-infected (NI) larvae maintained 100% survival over the 168 h period (data not shown). Larvae grown according to the IG methodology exhibited the lowest survival rate against the three pathogens. Although the TG showed the highest survival rates, no statistically significant difference was observed between the groups.

### 3.6 Effects of egg conditioning method on hemocyte recruitment

**Figure 6:**
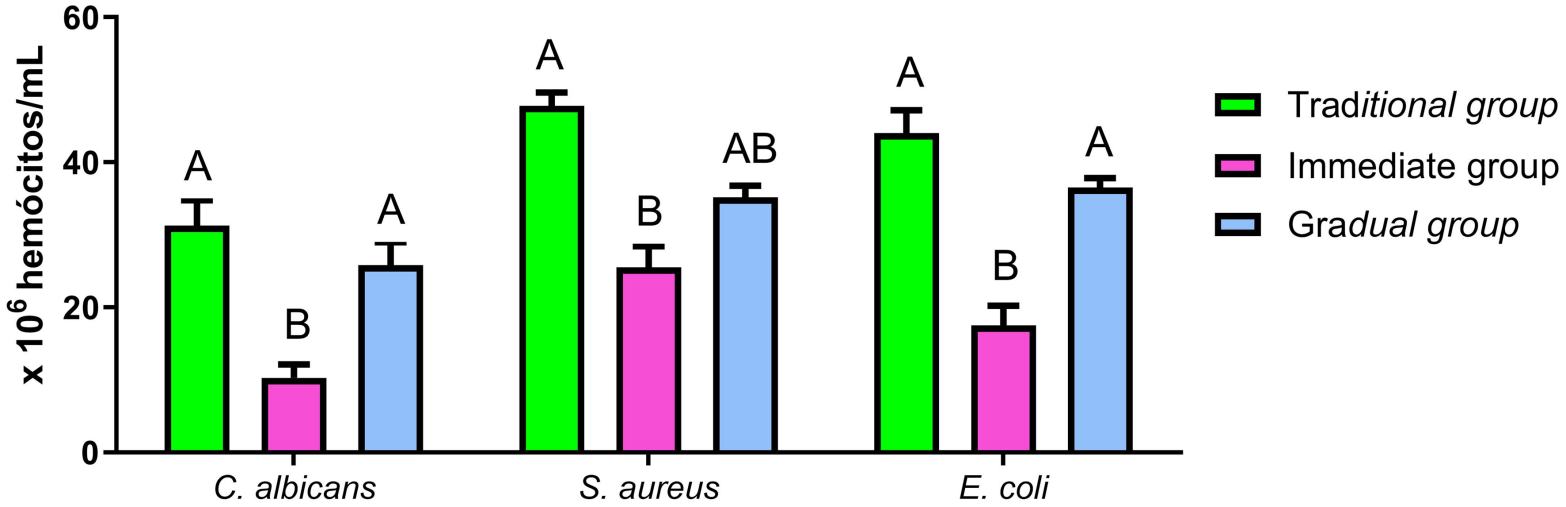
Hemocyte recruitment. Hemocyte recruitment after infection with *E. coli, C. albicans* and *S. aureus*: The hemolymph was recovered for the hemocyte count from the Traditional group (TG - green), Immediate group (IG - pink) and Gradual group (GG - blue). Statistical analyses were performed using one-way analysis of variance (ANOVA), followed by Tukey’s test. Different letters represent statistically significant differences (*p* < 0.05).

The concentration of hemocytes in the hemolymph was affected by the inoculated microorganism and the conditioning method. In *C. albicans* infection, the mean hemocyte count was 31.25 × 10^6^ hemocytes/mL, 10.25 × 10^6^ hemocytes/mL, and 25.83 × 10^6^ hemocytes/mL in TG, IG and GG, respectively. Hemocyte recruitment was 47.75 × 10^6^ hemocytes/mL for TG, 25.5 × 10^6^ hemocytes/mL in IG and 31.17 × 10^6^ hemocytes/mL for GG after infection with *E. coli*. Infection with *S. aureus* resulted in a hemocyte recruitment of 44 × 10^6^ hemocytes/mL, 17,5 × 10^6^ hemocytes/mL, and 36,5 × 10^6^ hemocytes/mL for TG, IG and GG, respectively (Figure 6). The IG presented significantly lower counts of hemocytes compared to the TG. In contrast, GG showed no statistically significant difference to TG, however differed from IG when inoculated with *C. albicans* and *E. coli*.

## 4 Discussion

The *G. mellonella* model has been widely used for studies in biomedical research (Gallorini et al., 2024; Benaducci et al., 2016; Lyu et al., 2017; Rossoni et al., 2017). However, maintaining this colony in the laboratory with consistent quality for research is challenging, mainly due to the need for continuous maintenance (Jorjão et al., 2018b; de Jong et al., 2022). In this study, we evaluated the effects of four egg conditioning methods (TG, IG, GG and FG) on the development and quality of the larvae produced. Furthermore, we studied the impact of these different conditioning methods on the susceptibility of larvae to infections by microorganisms (yeasts, and Gram-positive and -negative bacteria) and on the recruitment of hemocytes.

Viability analysis revealed that FG eggs failed to hatch throughout the entire experimental period (90 days), indicating a lack of viability after the applied conditioning and incubation methods. This finding is consistent with the practices of beekeepers, who use cold chambers or freezers set at temperatures between −7°C and −15°C for 2 to 4.5 h (h) to treat combs infested with *G. mellonella* eggs (Yadav and Kaushik, 2017). This method effectively transforms infested combs into marketable products (Kwadha et al., 2017). In contrast, TG, IG, and GG demonstrated egg hatching viability starting on day 7, following the stabilization of temperature and food conditions. Although the initial hatching among the groups was similar, there was a significant difference in the proportion of hatched eggs over time. The TG exhibited a more uniform hatching pattern, with most eggs hatching (≅95%) within a short period, corroborating the findings of Cardoso et al. (2007) who observed that eggs stored at 27 and 22°C maintained approximately 97% viability. On the other hand, IG and GG showed a broader distribution of hatching: GG reached a hatching rate of ≅95% within 28 days, while IG achieved ≅80% hatching within 21 days. This variation in hatching time is also observed in nature, where environmental factors can influence the hatching period, which may range from 3 to 30 days (Kwadha et al., 2017). Previous studies demonstrated a slowdown in metabolism and development of *G. mellonella* eggs and larvae stored at low temperatures (Cymborowski, 2000; Cardoso et al., 2007).

The storage conditions of FG impacted not only the hatching of the eggs but also the larval development. In the IG and GG, a heterogeneous larval profile, i.e., larvae at different stages of development, was observed at each time point. In contrast, TG exhibited homogeneous development, resulting in synchronous larval development. This lack of uniformity in larval stages can be a challenge for scientific research, which often requires many larvae at the same instar to ensure experimental standardization and reproducibility of results (Firacative et al., 2020).

During development, we observed healthy, active, uniform larvae, free of pigmentation signs and well-formed cocoons in the TG and GG larvae. The IG larvae also showed similar movement and cocoon formation to those in the TG and GG. However, these larvae exhibited significant melanization spots. The activation of the melanization pathways occurs inside the nodules in response to stressful stimuli, such as inadequate environmental conditions or bacterial or fungal infection (Gallorini et al., 2024; Guevara et al., 2022; Ten et al., 2023; Yu et al., 2024; Jothi and Gowrishankar, 2024). Furthermore, melanization serves as a disease marker and a parameter for determining the health index of larvae in survival curve analysis. Therefore, larvae showing signs of melanization should be discarded (Loh et al., 2013; Wang et al., 2023).

The developmental stages of *G. mellonella* can also be affected by temperature fluctuations. Roversi et al. (2008) demonstrated that eggs stored at temperatures below 20°C, even with cryoprotective agents, such as polyethylene glycol, experience a drastic reduction in viability. Indeed, we observed that eggs from FG did not hatch and larvae from IG exhibited a delayed development compared with larvae constantly maintained at 27°C (TG). Analyses of the larvae’s nervous system suggested that the arrest of larval development might be due to the inhibition of their prothoracic glands (Cymborowski, 2000). On the other hand, larvae usually do not spin cocoons or pupate at high temperatures (i.e., 40°C), probably due to hormonal imbalances (Stairs, 1978). In this study, the onset of pupation was slower in IG and GG as expected, with the first pupae observed on 49th and 70th days, respectively. In TG, the first pupa was observed on 42nd day, and all larvae had completed pupation after 49 days. The exclusive presence of pupae was observed on 63rd day in IG and on 77th day in GG. Altogether, these data demonstrate a delayed larval development and pupation in IG and GG compared with TG. This delay varied between 7 and 28 days for the emergence of the first pupa, and between 14 and 28 days for complete pupation of the group. A previous comparative study demonstrated that larvae of *G. mellonella* kept at 18°C exhibited reduced body weight and were unable to pupate (Cymborowski, 2000), corroborating the findings of this study. However, the larvae resumed development and successfully formed pupae when transferred to 30°C (Cymborowski, 2000). This result contrasts with the larvae in the present study, which, even when maintained at 27°C, showed slower metabolic activity and delayed development.

Therefore, we studied the effects of different conditioning and incubation protocols on the susceptibility of *G. mellonella* larvae to pathogens and on the recruitment of hemocytes. Although the survival curve showed no statistically significant differences, the IG exhibited the lowest survival rate following infection with all tested pathogens. Furthermore, hemocyte recruitment was significantly lower in the IG compared with TG when infected with *C. albicans*, *S. aureus*, or *E. coli*, and in the GG for infections with *C. albicans* and *E. coli*. The increase in the number of larval hemocytes is an important indicator of an active immune response, exhibiting protective effects against infection with *C. albicans* (de Barros et al., 2019; Rossoni et al., 2019), *S. aureus* (Sheehan et al., 2020; Alnezary et al., 2023) and *E. coli* (Jorjão et al., 2018a; Guerrieri et al., 2019). Hemocytes are cells found in the hemolymph that are responsible for the larva’s cellular immune response, which involves phagocytosis, encapsulation, and coagulation of foreign bodies, including pathogens (Jorjão et al., 2018b; Pereira and Rossi, 2020).

As observed in our study, changes in the storage temperature of *G. mellonella* eggs and larvae can also impact the susceptibility of the larvae to microbial pathogens. Browne et al. (2015) demonstrated that incubation of *G. mellonella* larvae at 15°C for up to 10 weeks reduced their survival in experimental infection with *C. albicans* and *S. aureus*. The increased susceptibility of the insect was related to a significant reduction in the hemocyte count, as well as in the enzymatic levels of phenoloxidase, the main effector of the melanization process. Conversely, shorter incubation periods (approximately 24 h) appear to have opposite outcomes to those of longer periods. *G. mellonella* larvae incubated at 4 or 37°C for 24 h prior to infection assays demonstrated reduced susceptibility to *C. albicans*. This phenotype was explained by an increase in hemocyte density and expression of the main antimicrobial peptides (AMPs), with a peak in expression of these peptides 24 h post-incubation (Mowlds and Kavanagh, 2008). Antimicrobial peptides (AMPs) are a group of proteins mainly produced in the body fat and display a broad-spectrum activity against bacteria, fungi, parasites, and viruses (Andrejko et al., 2021). In *G. mellonella*, AMPs such as gallerimycin and galiomycin play a fundamental role in survival against *C. albicans* infection (de Barros et al., 2021). Increased expression of the AMPs gloverin and cecropin has also been associated with enhanced immune protection during experimental *E. coli* infections (Leuko and Raivio, 2012). In addition, the synthetic AMP IKR18 has demonstrated significant anti-infective activity against *S. aureus*, including methicillin-resistant *Staphylococcus aureus* (MRSA) strains (Ramalho et al., 2022), highlighting the broad-spectrum antimicrobial potential of AMPs. The maintenance of larvae at 48°C for 24 h also increased the resistance of *G. mellonella* to infection by the fungal pathogen *Aspergillus fumigatus*, probably due to the increase in hemocyte recruitment, the expression of AMPs and the prophenoloxidase enzymatic activity. In contrast, longer incubation periods (48 to 72 h) resulted in a decrease in these protective factors for *G. mellonella* (Browne et al., 2014).

In summary, our findings demonstrated that different egg conditioning strategies directly affected the larval phenotype and hemocyte recruitment in *G. mellonella* larvae. While storage at low temperatures resulted in the absence of viability, abrupt temperature changes impaired larval development and hemocyte response. Surprisingly, gradual temperature conditioning methods preserved larval viability and immune function. Then, we suggest that, in cases where conventional rearing methodologies (such as those used in TG) are unfeasible, gradual and controlled temperature changes (as in GG) could be implemented. Among the methodologies tested, larvae from TG and GG exhibited comparable phenotype profiles, indicating similar responses under experimental conditions.

## Data Availability

The raw data supporting the conclusions of this article will be made available by the authors, without undue reservation.
